# Medical Officers under the Poor Law

**Published:** 1912-09-21

**Authors:** 


### Medical Officers Under the Poor Law.

THEIR NEW DUTIES UNDER THE PROPOSED ORDER.

A Departmental Committee of the Local Government Board, consisting of Sir J. S. Davy, Assistant Secretary of the Board; Sir S. B. Provis, ex-Permanent Secretary; Mr. W. T. Jerred, Assistant Secretary; Mr. Thomas Smith, Clerk to the West Ham Guardians; and Mr. H. W. S. Francis have, as we noted last week, issued a draft order for the government of Poor-Law institutions. We append below the suggestions which affect the status and duties of the medical officers of infirmaries, and which are contained in Article 57.

By it the medical officer shall (1) attend the inmates of the institution at the times fixed by the Guardians, when sent for in the manner provided by this order, and at such other times as may be necessary, and, where there is no assistant medical officer, name to the Guardians some qualified medical practitioner who will, in the case of the medical officer's absence, act in his place; (2) once at least in every month examine every child in the institution, report to the house committee any matters arising at such examination, and advise the house committee and the master as to any precautions which he may deem necessary for preventing the spread of infection; and examine every infant under the age of eighteen months as prescribed by Article 25; (3) examine every child whom it is proposed to transfer from the institution and certify the result, including the existence of any infectious disease; (4) as soon as practicable after the admission of a lunatic or alleged lunatic, and immediately before the removal of such to an institution, examine his person and enter in a book set apart for the purpose a statement of the condition of the inmate and of any bruises or marks of violence upon his person; (5) give to the superintendent nurse, or, if there be no superintendent nurse, to the matron, and to the nurses all necessary directions in regard to the treatment and nursing, and in any case of emergency report in writing to the master any need to obtain an additional nurse; (6) report to the Board within twenty-four hours —(*a*) every case of dangerous infectious disease, and (*b*) every sudden or accidental death, in respect of which an inquest is held, occurring among the inmates of the institution, except in the case of the death of a person who was not in receipt of relief at the time of the accident causing his death; (7) forthwith inform the master of every case of dangerous illness in the institution, and, immediately after the death of a lunatic or alleged lunatic, complete and furnish to the master a statement in the Form 18; (8) keep the record prescribed by Article 24 with respect to the seclusion of lunatics and alleged lunatics; (9) furnish to the medical officer of health for the district in which the institution is situate such information as the Board direct with respect to cases of sickness or death within the institution; (10) give to the house committee or the Guardians, when required, any reasonable information respecting any inmate, and certify in writing the sickness or other cause of his attendance on any inmate, on the request of the inmate or of any person acting on behalf of the inmate; (11) make whenever occasion arises, in a book set apart for the purpose and laid before the house committee at each meeting, reports, for the house committee and transmission to the Guardians, on (*a*) any matter affecting the health of the inmates; (*b*) any defects in the drainage, ventilation, warming, or other arrangements of the institution, and particularly of the sick and lunatic wards and the nurseries; (*c*) any defect in the nursing or attendance in the sick wards, the lunatic wards, or the nurseries, or in the supply of medical and surgical requirements; (*d*) any action taken by him under paragraph (5) of this Article; and (*e*) any other matter in which it is, in his opinion, desirable; (12) submit, for consideration and transmission to the Guardians, at the first meeting of the house committee after the first day of January and the first day of July in each year, a report of the condition of the institution; (13) in making entries in books, record-papers, or other records, employ the terms used in the regulations and statistical nosology issued by the Registrar-General.

It will be noted that the word workhouse is discarded, and the word pauper replaced by the less insulting term inmate. From Article 57 it will be seen that the draft order rescinds the provisions of the consolidated order in respect of the medical officer and other officials, and replaces them with other provisions.

